# Evaluating immunological and inflammatory changes of treatment-experienced people living with HIV switching from first-line triple cART regimens to DTG/3TC *vs.* B/F/TAF: the DEBATE trial

**DOI:** 10.3389/fimmu.2023.1279390

**Published:** 2023-10-16

**Authors:** Andrea Cossarizza, Alessandro Cozzi-Lepri, Marco Mattioli, Annamaria Paolini, Anita Neroni, Sara De Biasi, Domenico Lo Tartaro, Rebecca Borella, Lucia Fidanza, Lara Gibellini, Barbara Beghetto, Enrica Roncaglia, Giulia Nardini, Jovana Milic, Marianna Menozzi, Gianluca Cuomo, Margherita Digaetano, Gabriella Orlando, Vanni Borghi, Giovanni Guaraldi, Cristina Mussini

**Affiliations:** ^1^ Chair of Pathology and Immunology, University of Modena and Reggio Emilia School of Medicine, Modena, Italy; ^2^ Centre for Clinical Research, Epidemiology, Modelling and Evaluation (CREME), Institute for Global Health, University College London (UCL), London, United Kingdom; ^3^ Department of Surgical, Medical, Dental and Morphological Sciences, University of Modena and Reggio Emilia, Modena, Italy; ^4^ Clinic of Infectious Diseases, Azienda Ospedaliero-Universitaria Policlinico of Modena, Modena, Italy

**Keywords:** HIV, dual regimen, three-drug regimen, DTG/3TC, B/F/TAF, CD4/CD8 ratio

## Abstract

**Background:**

The aim of this randomized clinical trial (RCT) was to compare immunological changes in virally suppressed people living with HIV (PLWH) switching from a three-drug regimen (3DR) to a two-drug regimen (2DR).

**Methods:**

An open-label, prospective RCT enrolling PLWH receiving a 3DR who switched to bictegravir/emtricitabine/tenofovir alafenamide (B/F/TAF) or dolutegravir/lamivudine (DTG/3TC) was performed. Blood was taken at baseline and months 6 and 12. The primary outcome was the change in CD4+ or CD8+ T-cell counts and CD4/CD8 ratio over time points. The secondary outcomes were the changes in immunological and inflammatory parameters. Parametric mixed-linear models with random intercepts and slopes were fitted separately for each marker after controlling for potential confounders.

**Results:**

Between the two arms (33 PLWH each), there was no difference in CD4+ or CD8+ T cells, CD4/CD8 ratio, and IL-6 trajectories. PLWH switching to DTG/3TC had increased levels of both transitional memory and terminally differentiated CD4+ T cells (arm–time interaction p-value = 0.02) and to a lesser extent for the corresponding CD8+ T-cell subsets (p = 0.09). Significantly lower levels of non-classical monocytes were detected in the B/F/TAF arm at T6 (diff = −6.7 cells/mm^3^; 95% CI; −16, +2.6; p-value for interaction between arm and time = 0.03). All differences were attenuated at T12.

**Conclusion:**

No evidence for a difference in absolute CD4+ and CD8+ T-cell counts, CD4/CD8 ratio, and IL-6 trajectories by study arm over 12 months was found. PLWH on DTG/3TC showed higher levels of terminally differentiated and exhausted CD4+ and CD8+ T lymphocytes and non-classical monocytes at T6. Further studies are warranted to better understand the clinical impact of our results.

**Clinical Trial Registration:**

https://clinicaltrials.gov, identifier NCT04054089.

## Introduction

Bictegravir/emtricitabine/tenofovir alafenamide (B/F/TAF) is a guideline-recommended regimen for the treatment of HIV-1 infection in both naïve and experienced patients that demonstrated high efficacy and barrier to resistance without occurrence of incident resistance among individuals experiencing virological failure (1, 2). Dolutegravir/lamivudine (DTG/3TC) is the first dual antiretroviral therapy (ART) included by all international guidelines among recommended regimens for either ART-naïve or experienced people living with HIV (PLWH) (1, 2). This dual therapy (two-drug regimen (2DR)) has been evaluated in several trials in both ART-naïve (GEMINI 1-2) and virologically suppressed patients (TANGO and SALSA). In these large randomized clinical trials, DTG/3TC was shown to be non-inferior for virological outcomes to triple regimens (3DR), and this dual regimen is currently widely used in clinical practice (3–6).

Despite the lack of evidence for a difference in rates of virological suppression, whether levels of chronic inflammation differ after effective treatment with 2DR *vs.* 3DR remains to be established, and the question is increasingly relevant due to the introduction of cabotegravir/rilpivirine long-acting 2DR in routine clinical practice. In order to test this hypothesis, several studies have been conducted to evaluate differences in viral replication efficacy, viral reservoir, or inflammatory biomarkers.

Concerning viral replication, DTG/3TC in either naïve or experienced patients was shown to be non-inferior to 3DRs for the outcomes of target not detectable viremia or viral blips (7, 8). Moreover, a recent sub-study of the RUMBA trial did not show any evidence at 48 weeks for a difference in HIV-1 reservoir between people treated with DTG/3TC and those receiving B/F/TAF (9).

Concerning chronic inflammation, the TANGO randomized trial, comparing PLWH switching to DTG/3TC to those remaining on a 3DR, has shown a statistically significant 16% increase in interleukin (IL)-6 plasma levels at 48 weeks in the first group (4). A similar increase in both IL-6 and D-dimer was also observed in the CoRIS observational cohort, detected only after 3 years of switching to different 2DRs (10).

The aim of this randomized clinical trial was to compare the immunophenotype and inflammatory biomarkers in PLWH with viral load ≤50 copies/mL switching from a 3DR to B/F/TAF *vs.* DTG/3TC.

## Methods

This is an open-label, prospective, single-center, randomized trial enrolling PLWH seen for care at the Infectious Disease Clinic of Azienda Ospedaliero-Universitaria Policlinico of Modena, Italy. The study, with EudraCT Number 2018-003458-26, was approved by the local Ethical Committee with Authorization Number AOU 0010923/19 on April 4, 2019, and AIFA Authorization Number AIFA/SC/P/33830 on March 25, 2019.

Participants who had a suppressed HIV-RNA ≤50 copies/mL on a 3DR ART regimen for >12 months without any previous failure (previous switches to any other 3DRs with HIV-RNA ≤50 copies/mL were allowed), after providing written informed consent, were randomized 1:1 by means of simple randomization either to switch to B/F/TAF or DTG/3TC and were subsequently followed up for 48 weeks. Concealment of allocation was guaranteed using opaque envelopes so that random assignment was not revealed to the recruiters until participants had passed all screening tests and were deemed to be eligible. Participants have been enrolled over a period of 18 months. Other key inclusion criteria were as follows: i) age ≥18 years; ii) the ability to understand and sign a written informed consent form, which must be obtained prior to initiation of study procedures; and iii) no history of viral blips or virological failure prior to enrolment. Virological failure after randomization was defined as a confirmed virological rebound of HIV-1 RNA ≥ 50 copies/mL at two consecutive visits. Since at the time of enrollment there were still concerns about dolutegravir safety in pregnancy, female participants who were pregnant or planning pregnancy were excluded. More details about inclusion and exclusion criteria are included in Supplementary Annex 1.

### Outcomes

The primary outcome of the trial was the change in CD4+ or CD8+ T-cell counts and CD4/CD8 ratio from enrolment to week 48.

The secondary outcomes were the changes in inflammation and other immune characteristics (phenotype of T cells, proportion of cells belonging to different CD4+ and CD8+ T-cell subsets, monocytes, and plasma levels of IL-6) from enrolment to week 48.

### Immunological analyses

Immunological analyses are described in detail in Supplementary Annex 2 (11–16).

### Statistical analyses

Participants’ characteristics by study arm were described and reported as the number of participants with relative frequencies for categorical factors and as the median and interquartile range (IQR) for continuous variables.

Repeated measurements of all biomarkers were available at the three fixed time points (T0 = baseline, T6 = 6 months after randomization, and T12 = 12 months after randomization) of the trial overall, and the description was broken down by study arm. Box plots were used to describe the distribution of the raw data at the three time points (the graphs display values below Q1 and Q3 as outliers). We then performed unadjusted and adjusted parametric mixed-linear models with random intercepts and slopes including the main effects (study arm and time) as well as the interaction term. The adjusted model also included the key baseline confounders age, nationality, AIDS, duration of HIV, duration of viral load (VL) suppression, and CD4 count nadir. We fitted separate models for each of the outcome variables in the log scale and estimated at each time point the mean levels and difference by study arm with the corresponding 95% CI using the model predictions.

Two symmetric analyses were conducted: the first using as the main outcome the absolute parameters values and the second analysis using the changes from T0 as an alternative outcome. Although the two approaches and the related statistical tests partially overlap statistically, this second analysis allows the estimation of the parameter changes from T1 by study arm. Specific contrasts (i.e., the mean CD4 count difference by study arm at T6) were considered only when there was an overall type 3 significant p-value (<0.05) for the global test for interaction between study arm and time. All participants stayed on the randomization treatment until the end of the study. Although a total of 10 parameters (CD4, CD8, ratio, CD4 TM, CD4 EMRA, CD8 TM, CD8 EMRA, two classes of monocytes, and IL-6) were fitted, these analyses were conceived *a priori* before seeing the data. Further details regarding the fit of the regression model and checking of assumptions are reported in Supplementary Annex 3. All the above-mentioned analyses were performed by SAS version 9.4 (Carey, NC, USA), Prism 6.0 (GraphPad, La Jolla, CA, USA), and STATA 13.0 (College Station, TX, USA) software.

### Sample size calculations

We based the sample size calculations on the primary outcome change in CD8 count from T0 to T12. At the time of writing the trial protocol, we hypothesized that switching to DTG/3TC could be associated with a higher CD8 activation compared to switching to B/F/TAF. In particular, on the basis of data previously published in the literature available at the time of the protocol, we assumed that CD8 count could increase by 20 cells/mm^3^ in the DTG/3TC switching strategy *vs.* 15 cells/mm^3^ in the B/F/TAF switching strategy by week 48 (i.e., a difference of 5 cells/mm^3^). At a common STD = 5, we estimated that n = 66 patients (33 per arm) would be needed to detect this difference with a power of 81% after fixing the type I error to the standard threshold of 0.05 (two-sided test) (11). Because of the short, planned follow-up, we did not adjust these estimates for attrition.

## Results

Between September 2020 and January 2021, 66 patients attending the Clinic of Infectious Diseases of the Azienda Ospedaliero-Universitaria Policlinico of Modena were enrolled in the trial. Epidemiological characteristics are described in **Table 1**. In particular, 86% (n = 57) were male, the median age was 54 years (IQR: 43, 59), and the participants were mostly Italians. The median duration of known HIV infection was 14 years (IQR: 9, 20), and the median duration of plasma viral load ≤50 copies/mL was 101 months (IQR: 72, 161). Approximately 14% (n = 9) had a diagnosis of AIDS prior to enrolment. The median nadir CD4 lymphocyte count was 296 cells/µL (IQR: 197, 375), while the median baseline CD4 lymphocyte count was 768 cells/µL (IQR: 581, 1,070) (**Table 1**).

**Table 1:** Main characteristics of target population by study arm.

	DEBATE arm		
Characteristics	DTG/3TC	B/F/TAF	Total
	N = 33	N = 33	N = 66
Age, years			
Median (IQR)	48 (43, 57)	55 (43, 60)	54 (43, 59)
Sex at birth, n (%)			
Male	29 (87.9%)	28 (84.8%)	57 (86.4%)
Nationality, n (%)			
Foreign	6 (18.2%)	3 (9.1%)	9 (13.6%)
Mode of HIV transmission, n (%)			
Heterosexual contacts	9 (27.3%)	10 (30.3%)	19 (28.8%)
MSM	19 (57.6%)	16 (48.5%)	35 (53.0%)
PWID	2 (6.1%)	6 (18.2%)	8 (12.1%)
Other	3 (9.1%)	1 (3.0%)	4 (6.1%)
Duration of known HIV infection, years			
Median (IQR)	12 (8, 17)	16 (12, 22)	14 (9, 20)
Time with VL below 50 cps/mL, months			
Median (IQR)	98 (73, 139)	120 (71, 170)	101 (72, 161)
***HCVAb+, n (%)***	6 (19.4%)	4 (12.5%)	10 (15.9%)
***Previous AIDS, n (%)***	6 (18.2%)	3 (9.1%)	9 (13.6%)
CD4 and CD8 count, median (IQR)			
CD4 nadir	303 (183, 413)	268 (208, 354)	296 (197, 375)
CD4 baseline	734 (543, 971)	809 (648, 1,084)	768 (581, 1,070)
CD4/CD8 ratio	1.0 (0.6, 1.3)	1.1 (0.8, 1.3)	1.0 (0.7, 1.3)
3rd agent in previous therapy, n (%)			
*INSTI*	10 (30.3%)	9 (27.3%)	19 (28.8%)
RAL	1 (3.0%)	2 (6.1%)	3 (4.5%)
DTG	5 (15.2%)	4 (12.1%)	9 (13.6%)
EVG	4 (12.1%)	3 (9.1%)	7 (10.6%)
*PI/r*	0 (0.0%)	5 (15.2%)	5 (7.6%)
ATV	0 (0.0%)	2 (6.1%)	2 (3.0%)
DRV	0 (0.0%)	3 (9.1%)	3 (4.5%)
*NNRTI*	22 (66.7%)	19 (57.6%)	41 (62.1%)
EFV	8 (24.2%)	3 (9.1%)	11 (16.7%)
NVP	2 (6.1%)	5 (15.2%)	7 (10.6%)
RPV	12 (36.4%)	11 (33.3%)	23 (34.8%)
*NRTI*	1 (3.0%)	0 (0.0%)	1 (1.5%)
NRTI backbone			
ABC+3TC	8 (24.2%)	9 (27.3%)	17 (25.8%)
FTC+TDF	8 (24.2%)	6 (18.2%)	14 (21.2%)
FTC+TAF	16 (48.5%)	17 (51.5%)	33 (50.0%)
AZT+3TC	1 (3.0%)	1 (3.0%)	2 (3.0%)
Other biochemical, median (IQR)			
Weight, kg	78.0 (64.0, 85.0)	76.0 (69.0, 87.0)	78.0 (68.0, 87.0)
BMI, kg/m^2^	24.7 (22.3, 29.1)	25.5 (23.7, 29.2)	25.3 (22.8, 29.1)
CD8 count, cells/mm^3^	38.4 (33.6, 46.8)	34.8 (29.9, 39.5)	36.6 (31.1, 44.7)
Glucose, mg/dL	89.0 (79.0, 96.0)	89.0 (83.0, 94.0)	89.0 (81.0, 96.0)
Triglycerides, mg/dL	112.5 (80.0, 149.0)	104.5 (79.0, 154.0)	110.5 (79.0, 154.0)
Total cholesterol, mg/dL	181.0 (168.0, 215.0)	185.0 (168.0, 212.0)	182.0 (168.0, 215.0)
LDL cholesterol, mg/dL	112.0 (106.0, 141.0)	107.0 (97.0, 126.0)	109.5 (98.5, 128.0)
HDL cholesterol, mg/dL	50.0 (49.0, 55.0)	57.0 (43.0, 67.0)	53.5 (45.5, 64.0)
Total-to-HDL cholesterol ratio	4.0 (3.1, 4.3)	3.3 (2.0, 4.0)	3.6 (2.6, 4.1)
Creatinine, mg/dL	0.9 (0.9, 1.1)	1.0 (0.8, 1.1)	0.9 (0.8, 1.1)
eGFR, mL/min per 1·73 m^2^	92.9 (77.7, 101.4)	89.2 (74.9, 97.6)	91.1 (77.3, 101.1)
Proteinuria, mg/g	0.1 (0.1, 10.0)	0.1 (0.1, 0.1)	0.1 (0.1, 0.1)

IQR, interquartile range; MSM, men who have sex with men; PWID, people who inject drugs; LDL, low-density lipoprotein; HDL, high-density lipoprotein; eGFR, estimated glomerular filtration rate.

At baseline, patients were randomized to switch either to DTG/3TC (n = 33) or to B/F/TAF (n = 33). Despite randomization, the two groups differed in the proportion of those who were previously diagnosed with AIDS (18.2% DTG/3TC n = 6 *vs.* 9.1% B/F/TAF n = 3), but the absolute number of participants with previous AIDS was very low (6 patients *vs.* 3), and nadir CD4 count also tended to be higher in the dual regimen (303 *vs.* 268 cell/µL). Levels of CD4 at baseline and CD4/CD8 ratio at baseline were similar. There was, however, a large imbalance in other demographics such as age (younger in those randomized to the dual regimen) and nationality (twofold higher frequency of participants of foreign nationality in DTG/3TC). Finally, the duration of HIV infection and time spent with an undetectable plasma viral load were slightly longer in the B/F/TAF arm (120 weeks compared to 98 weeks). In the longitudinal analysis, 33 patients in DTG/3TC and 31/33 in BIC were included because shortly after randomization, one discontinued due to rash and one due to lymphatic leukemia. For one participant in the DTG/3TC arm, the blood sample could not be analyzed at T0 for technical reasons (**Table 1**).

### Primary outcomes: CD4+ or CD8+ T-cell count and CD4/CD8 ratio

Concerning the primary endpoint of absolute values and change in CD4+ or CD8+ T lymphocyte count and in CD4/CD8 ratio, no evidence for a difference by study arm was found either at 6 (T6) or at 12 months (T12) after the switch (T0). All these three main immunological parameters tended to remain stable over time in both arms (**Table 2A**, **Supplementary Figure 1**). In the adjusted model, after controlling for detected baseline imbalances in age, nationality, previous AIDS, duration of HIV, duration of VL suppression, and CD4 count nadir, there was no evidence for a difference in trajectories by study arm over time (p = 0.30 for CD4 count, p = 0.38 for CD8+ T-cell count, and p = 0.73 for the CD4/CD8 ratio). For CD4+ T-cell count, participants allocated to the B/F/TAF arm had a higher count than those to the DTG/3TC arm at T0, and this difference remained stable over time. A similar trend was seen for CD8+ T cells and the CD4/CD8 ratio, although the magnitude of the difference was smaller and even more stable over time. Similar results were obtained when the slopes (change from T0 values) instead of the absolute values were modeled as shown in **Table 2B** and **Supplementary Figure 1**.

**Table 2A:** Primary endpoints by study arm—adjusted analysis.

	Visits							
	Adjusted^&^ means			Adjusted^&^ difference in means				
Markers								
	T0 95% CI	T6 95% CI	T12 95% CI	T0 95% CI p-Value	T6 95% CI p-Value		T12 95% CI p-Value	p-Value^*^
CD4 count, cells/mm^3^								
***RCT arm***								0.297
DTG/3TC	800 (696, 905)	818 (714, 922)	754 (650, 858)	0	0		0	
B/F/TAF	943 (834, 1,053)	905 (795, 1,014)	906 (797, 1,016)	143 (−13, 299)	87 (−69, 243)		152 (−4, 309)	
				0.076	0.277		0.058	
CD8 count, cells/mm^3^								
***RCT arm***								0.380
DTG/3TC	908 (766, 1,050)	931 (790, 1,072)	853 (712, 995)	0	0		0	
B/F/TAF	938 (789, 1,087)	898 (749, 1,047)	880 (731, 1,029)	30 (−183, 243)	−33 (−245, 180)		27 (−186, 239)	
				0.782	0.762		0.807	
CD4/CD8 ratio, units								
***RCT arm***								0.731
DTG/3TC	1.0 (0.8, 1.1)	1.0 (0.8, 1.2)	1.0 (0.8, 1.2)	0	0	0		
B/F/TAF	1.1 (0.9, 1.3)	1.1 (0.9, 1.3)	1.1 (1.0, 1.3)	0.1 (−0.1, 0.4)	0.1 (−0.2, 0.4)	0.1 (−0.1, 0.4)		
				0.414	0.451	0.299		

RCT, randomized clinical trial; VL, viral load.

^*^F-test type 3 interaction p-value.

^&^Adjusted for age, nationality, AIDS, duration of HIV, duration of VL suppression, and CD4 count nadir.

**Table 2B:** Primary endpoints (change) by study arm—adjusted analysis.

	Visits						
	Adjusted^&^ mean changes from T0			Adjusted^&^ difference in mean changes from T0			
Markers							
	T0 95% CI	T6 95% CI	T12 95% CI	T0 95% CI p-Value	T6 95% CI p-Value	T12 95% CI p-Value	p-Value^*^
CD4 count, cells/mm^3^							
***RCT arm***							0.348
DTG/3TC	−8 (−64, 49)	12 (−45, 68)	−53 (−110, 4)	0	0	0	
B/F/TAF	8 (−50, 67)	−30 (−89, 29)	−29 (−87, 30)		−41 (−125, 42)	24 (−59, 108)	
					0.330	0.564	
CD8 count, cells/mm^3^							
***RCT arm***							0.457
DTG/3TC	−12 (−79, 56)	13 (−55, 80)	−62 (−129, 6)	0	0	0	
B/F/TAF	13 (−57, 82)	−27 (−97, 42)	−46 (−115, 24)		−40 (−139, 59)	16 (−83, 115)	
					0.428	0.751	
CD4/CD8 ratio, units							
***RCT arm***							0.626
DTG/3TC	0.0 (−0.1, 0.1)	0.0 (−0.0, 0.1)	0.0 (−0.0, 0.1)	0	0	0	
B/F/TAF	−0.0 (−0.1, 0.1)	0.0 (−0.1, 0.1)	0.1 (−0.0, 0.1)		−0.0 (−0.1, 0.1)	0.0 (−0.1, 0.1)	
					0.717	0.531	

RCT, randomized clinical trial; VL, viral load.

^*^ F-test type 3 interaction p-value.

^&^ Adjusted for age, nationality, AIDS, duration of HIV, duration of VL suppression, and CD4 count nadir.

### CD4+ T-cell subsets

**Figure 1:**
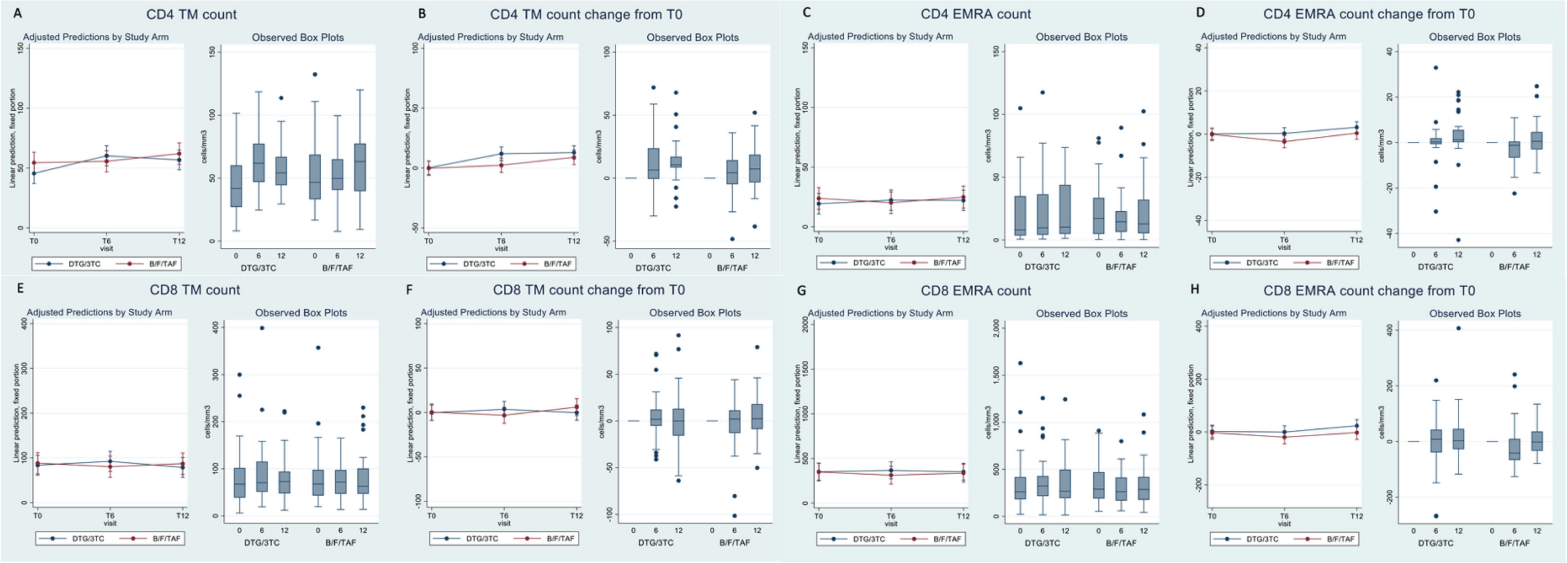
Box plots of CD4 and CD8 count subsets and adjusted prediction with 95% CI from fitting the mixed-linear model.

In contrast, when we looked at the proportion of CD4+ T-cell subsets, significant differences in trends by arm were detected for both TM and EMRA (arm–time interaction p-value = 0.02 in the adjusted analysis). The significance was likely to be driven by the trends at T6, the time at which values of both TM (−4.6 cells/mm^3^; 95% CI: −17.1, +8.0) and EMRA (−2.0 cells/mm^3^; 95% CI: −14.9, +10.9) tended to be higher for participants in DTG/3TC *vs.* B/F/TAF. However, despite the overall significance, there was a large uncertainty in the estimates from the mixed model as illustrated by the large confidence intervals. This difference tended to be again attenuated at time T12, and data appeared to be similar to those seen at entry in the trial (**Table 3A**, **Figure 1**). Again, similar results were observed when we looked at the output of the model when the change from T0 was fitted instead of the absolute values (**Table 3A**, **Figure 1**).

**Table 3A:** CD4 subsets by study arm—adjusted analysis.

	Visits						
	Adjusted means			Adjusted difference in means			
Markers	T0 95% CI	T6 95% CI	T12 95% CI	T0 95% CI p-Value	T6 95% CI p-Value	T12 95% CI p-Value	p-Value^*^
CD4 TM, cells/mm^3^							
***RCT arm***							0.019
DTG/3TC	45.4 (37.1, 53.8)	60.2 (51.8, 68.5)	56.7 (48.5, 65.0)	0	0	0	
B/F/TAF	54.4 (45.6, 63.3)	55.6 (46.8, 64.5)	62.2 (53.2, 71.2)	9.0 (−3.5, 21.5)	−4.6 (−17.1, 8.0)	5.4 (−7.1, 18.0)	
				0.161	0.477	0.399	
CD4 EMRA, cells/mm^3^							
***RCT arm***							0.019
DTG/3TC	19.3 (10.8, 27.8)	22.3 (13.8, 30.8)	22.0 (13.6, 30.5)	0	0	0	
B/F/TAF	23.8 (14.8, 32.9)	20.2 (11.2, 29.3)	24.8 (15.7, 33.9)	4.6 (−8.3, 17.5)	−2.0 (−14.9, 10.9)	2.7 (−10.2, 15.6)	
				0.486	0.758	0.680	
CD4 CD57 PD1, cells/mm^3^							
***RCT arm***							0.159
DTG/3TC	11.3 (7.0, 15.5)	15.7 (11.5, 20.0)	15.8 (11.6, 20.0)	0	0	0	
B/F/TAF	15.1 (10.6, 19.6)	15.6 (11.1, 20.1)	19.0 (14.5, 23.6)	3.8 (−2.6, 10.1)	−0.1 (−6.5, 6.2)	3.2 (−3.2, 9.6)	
				0.248	0.970	0.323	
CD4 subsets (change) by study arm—adjusted analysis.							
	Visits						
	Adjusted mean changes from T0			Adjusted difference in mean changes from T0			
Markers							
	**T0** **95% CI**	**T6** **95% CI**	**T12** **95% CI**	**T0** **95% CI** **p-Value**	**T6** **95% CI** **p-Value**	**T12** **95% CI** **p-Value**	p-Value^*^
CD4 TM, cells/mm^3^							
***RCT arm***							0.066
DTG/3TC	0.1 (−5.4, 5.7)	11.9 (6.4, 17.5)	13.0 (7.5, 18.4)	0	0	0	
B/F/TAF	−0.2 (−6.1, 5.6)	2.3 (−3.6, 8.2)	9.0 (3.1, 15.0)		−9.6 (−17.9, −1.3)	−3.9 (−12.3, 4.4)	
					0.025	0.358	
CD4 EMRA, cells/mm^3^							
***RCT arm***							0.285
DTG/3TC	0.1 (−2.5, 2.8)	0.4 (−2.3, 3.0)	3.2 (0.5, 5.8)	0	0	0	
B/F/TAF	−0.1 (−2.9, 2.7)	−3.5 (−6.3, −0.7)	0.4 (−2.4, 3.3)		−3.8 (−7.8, 0.1)	−2.7 (−6.7, 1.2)	
					0.058	0.179	
CD4 CD57 PD1, cells/mm^3^							
***RCT arm***							0.366
DTG/3TC	−0.1 (−2.5, 2.4)	3.5 (1.0, 5.9)	4.8 (2.3, 7.2)	0	0	0	
B/F/TAF	0.1 (−2.5, 2.7)	0.7 (−1.9, 3.3)	3.6 (1.0, 6.3)		−2.8 (−6.4, 0.9)	−1.2 (−4.8, 2.5)	
					0.144	0.543	

RCT, randomized clinical trial; VL, viral load.

^*^ F-test type 3 interaction p-value.

^&^ Adjusted for age, nationality, AIDS, duration of HIV, duration of VL suppression, and CD4 count nadir.

### CD8+ T-cell subsets

The results for the CD8+ T-cell subsets were similar to those seen for the corresponding CD4+ T cells shown above, although none of the statistical interaction tests reached statistical significance (p = 0.09 for TM and p = 0.19 for EMRA in the adjusted analysis, **Table 3B**, **Figure 1**). The differences at T0 between DTG/3TC and B/F/TAF were in the same direction (higher values in DTG/3TC *vs.* 3DR), although the magnitude in terms of cells/µL was even bigger compared to the measures in the equivalent CD4+ T-cell subset, still with large uncertainty around these estimates.

**Table 3B:** CD8 subsets by study arm—adjusted analysis.

	Visits						
	Adjusted means			Adjusted difference in means			
Markers							
	T0 95% CI	T6 95% CI	T12 95% CI	T0 95% CI p-Value	T6 95% CI p-Value	T12 95% CI p-Value	p-Value^*^
CD8 TM, cells/mm^3^							
***RCT arm***							0.086
DTG/3TC	83.7 (61.5, 106.0)	92.3 (70.1, 114.5)	78.8 (56.7, 100.9)	0	0	0	
B/F/TAF	87.8 (64.2, 111.4)	80.0 (56.4, 103.6)	86.8 (63.0, 110.7)	4.1 (−29.4, 37.5)	−12.3 (−45.8, 21.2)	8.0 (−25.6, 41.6)	
				0.812	0.473	0.640	
CD8 EMRA, cells/mm^3^							
***RCT arm***							0.186
DTG/3TC	351.2 (254.7, 447.6)	364.6 (268.2, 461.0)	349.4 (253.1, 445.6)	0	0	0	
B/F/TAF	350.2 (247.3, 453.0)	314.7 (211.8, 417.6)	336.2 (232.9, 439.5)	−1.0 (−147, 145.2)	−49.9 (−196, 96.3)	−13.2 (−160, 133.3)	
				0.990	0.505	0.860	
CD8 CD57 PD1, cells/mm^3^							
***RCT arm***							0.160
DTG/3TC	14.7 (9.1, 20.2)	19.0 (13.5, 24.6)	20.1 (14.6, 25.6)	0	0	0	
B/F/TAF	16.1 (10.2, 22.0)	15.9 (10.0, 21.8)	20.3 (14.4, 26.3)	1.4 (−6.9, 9.8)	−3.1 (−11.5, 5.2)	0.2 (−8.1, 8.6)	
				0.739	0.461	0.955	
CD8 subsets (change) by study arm—adjusted analysis.							
	Visits						
	Adjusted mean changes from T0			Adjusted difference in mean changes from T0			
Markers							
	T0 95% CI	T6 95% CI	T12 95% CI	T0 95% CI p-Value	T6 95% CI p-Value	T12 95% CI p-Value	p-Value^*^
CD8 TM, cells/mm^3^							
***RCT arm***							0.212
DTG/3TC	−0.3 (−9.0, 8.3)	3.6 (−5.1, 12.3)	−0.2 (−8.7, 8.4)	0	0	0	
B/F/TAF	0.4 (−8.8, 9.5)	−3.1 (−12.3, 6.0)	6.1 (−3.2, 15.4)		−6.7 (−19.6, 6.1)	6.3 (−6.6, 19.2)	
					0.306	0.341	
CD8 EMRA, cells/mm^3^							
***RCT arm***							0.673
DTG/3TC	2.2 (−21.3, 25.6)	−0.7 (−24.3, 22.8)	23.3 (0.1, 46.5)	0	0	0	
B/F/TAF	−2.6 (−27.4, 22.1)	−19.2 (−44.0, 5.5)	−2.3 (−27.6, 23.0)		−18.5 (−53.3, 16.3)	−25.6 (−60.6, 9.4)	
					0.299	0.155	
CD8 CD57 PD1, cells/mm^3^							
***RCT arm***							0.200
DTG/3TC	−0.1 (−3.0, 2.8)	4.1 (1.2, 7.0)	6.3 (3.4, 9.2)	0	0	0	
B/F/TAF	0.1 (−3.0, 3.2)	−0.0 (−3.1, 3.0)	4.0 (0.8, 7.1)		−4.1 (−8.5, 0.2)	−2.3 (−6.7, 2.0)	
					0.064	0.299	

^*^ F-test type 3 interaction p-value.

^&^ Adjusted for age, nationality, AIDS, duration of HIV, duration of VL suppression, and CD4 count nadir.

RCT, randomized clinical trial; VL, viral load.

^*^ F-test type 3 interaction p-value.

^&^ Adjusted for age, nationality, AIDS, duration of HIV, duration of VL suppression, and CD8 count nadir.

### Monocytes

We then investigated trend in monocytes, specifically by distinguishing between classical and non-classical subsets (note that non-classical monocytes also included a small proportion of intermediate monocytes). Although no statistical significance was detected for the classical monocyte subsets (interaction p-value=0.50), for the non-classical monocytes we detected a strong association by arm and time (interaction p-value=0.03 in the adjusted model). Also in this analysis, the significance was likely driven by the difference at T6 when non-classical monocytes were significantly lower in B/F/TAF vs the DTG/3TC arm (diff =-6.7 cells/mm3, 95% CI; -16;+2.6). The difference was again attenuated at T12 (**Table 4A**, **Supplementary Figure 2**).

**Table 4A:** Monocytes by study arm—adjusted analysis.

	Visits						
	Adjusted means			Adjusted difference in means			
Markers	T0 95% CI	T6 95% CI	T12 95% CI	T0 95% CI p-Value	T6 95% CI p-Value	T12 95% CI p-Value	p-Value^*^
Classical Monocytes, cells/mm^3^							
***RCT arm***							0.498
DTG/3TC	335.9 (293.7, 378.2)	365.9 (323.7, 408.1)	352.4 (310.1, 394.6)	0	0	0	
B/F/TAF	372.6 (327.7, 417.4)	384.7 (339.9, 429.5)	387.3 (342.1, 432.5)	36.6 (−27.1, 100.3)	18.8 (−44.8, 82.5)	35.0 (−29.0, 99.0)	
				0.262	0.563	0.286	
Non-classical Monocytes, cells/mm^3^							
***RCT arm***							0.030
DTG/3TC	28.2 (21.9, 34.5)	36.5 (30.2, 42.8)	38.4 (32.1, 44.7)	0	0	0	
B/F/TAF	35.1 (28.5, 41.7)	29.8 (23.2, 36.4)	38.1 (31.4, 44.9)	6.9 (−2.4, 16.2)	−6.7 (−16.0, 2.6)	−0.3 (−9.7, 9.1)	
				0.151	0.163	0.952	
Monocytes (change) by study arm—adjusted analysis.							
	Visits						
	Adjusted means			Adjusted difference in means			
Markers	T0 95% CI	T6 95% CI	T12 95% CI	T0 95% CI p-Value	T6 95% CI p-Value	T12 95% CI p-Value	p-Value^*^
Classical Monocytes, cells/mm^3^							
***RCT arm***							0.584
DTG/3TC	−1.1 (−25.1, 22.9)	28.9 (4.7, 53.2)	15.1 (−9.2, 39.3)	0	0	0	
B/F/TAF	1.4 (−23.8, 26.7)	13.6 (−11.6, 38.8)	13.9 (−11.8, 39.6)		−15.3 (−51.1, 20.4)	−1.1 (−37.3, 35.0)	
					0.402	0.951	
Non-classical Monocytes, cells/mm^3^							
***RCT arm***							0.025
DTG/3TC	−0.7 (−6.6, 5.2)	7.7 (1.7, 13.7)	9.8 (3.9, 15.8)	0	0	0	
B/F/TAF	0.7 (−5.5, 6.9)	−4.6 (−10.8, 1.6)	4.0 (−2.3, 10.3)		−12.3 (−21.0, −3.5)	−5.8 (−14.7, 3.0)	
					0.007	0.198	

RCT, randomized clinical trial.

### Inflammation: IL-6

We finally analyzed the trend in IL-6, chosen as the most important cytokine related to onset and maintenance of inflammation. Because of the trend seen with the CD4+ and CD8+ TM/EMRA T cell subsets (large difference by study arm at T6 followed by an attenuation of the difference at T12), we hypothesized that this might have been caused by a return to normal in the overall level of inflammation at T6. However, the data carried no evidence of a difference in the trajectories of IL-6 over T0-T12 by study arm, and especially the difference by arm in IL-6 tended to remain small and stable over T6-T12 (**Table 4B**, **Supplementary Figure 2**, 0.4 pg/mL difference, p-value for interaction =0.49 in the adjusted analysis).

**Table 4B:** IL-6 by study arm—adjusted analysis.

	Visits						
	Unadjusted means			Unadjusted difference in means			
Markers	T0 95% CI	T6 95% CI	T12 95% CI	T0 95% CI p-Value	T6 95% CI p-Value	T12 95% CI p-Value	p-Value^*^
IL-6, pg/mL							
***RCT arm***							0.487
DTG/3TC	2.6 (1.9, 3.3)	2.7 (2.0, 3.4)	2.5 (1.8, 3.2)	0	0	0	
B/F/TAF	2.4 (1.7, 3.1)	3.1 (2.3, 3.8)	3.0 (2.3, 3.7)	−0.2 (−1.2, 0.8)	0.4 (−0.6, 1.4)	0.5 (−0.5, 1.5)	
				0.728	0.484	0.326	
IL-6 (change) by study arm—adjusted analysis.							
	Visits						
	Adjusted means			Adjusted difference in means			
Markers	T0 95% CI	T6 95% CI	T12 95% CI	T0 95% CI p-Value	T6 95% CI p-Value	T12 95% CI p-Value	p-Value^*^
IL-6, pg/mL							
***RCT arm***							0.504
DTG/3TC	0.0 (−0.6, 0.6)	0.2 (−0.4, 0.8)	−0.0 (−0.7, 0.6)	0	0	0	
B/F/TAF	−0.0 (−0.7, 0.6)	0.6 (−0.0, 1.3)	0.6 (−0.1, 1.3)	−0.0 (−1.0, 0.9)	0.4 (−0.5, 1.3)	0.6 (−0.3, 1.6)	
				0.931	0.399	0.180	

^*^ F-test type 3 interaction p-value.

RCT, randomized clinical trial; VL, viral load.

^*^ F-test type 3 interaction p-value.

^&^ Adjusted for age, nationality, AIDS, duration of HIV, duration of VL suppression, and CD4 count nadir.

## Discussion

Our randomized study shows no evidence for a difference in absolute CD4+ and CD8+ T-cell counts and in CD4/CD8 ratio trajectories over 12 months of follow-up by study arm (DTG/3TC *vs.* B/F/TAF). These data are in disagreement with those coming from a previous analysis of the ICONA cohort data (12), but consistent with others coming from real-world studies with one that had shown even an increase in CD4/CD8 ratio after the switch to DTG/3TC (over an average follow-up of 12–60 months) (12).

However, importantly, our data provide additional insights concerning immunological changes after switching from 3DR treatment to DTG/3TC or B/F/TAF. First, the increase in CD4+ and to a lesser extent in CD8+ activated T cells, coupled with an increase in transitional memory and terminally differentiated cells, especially at 6 months after switching to DTG/3TC, but not after switching to B/F/TAF, could indicate an effect of DTG/3TC, which goes beyond reaching an undetectable plasma viral load. Second, in order to synthesize soluble or plasma membrane molecules that provide signals to the cells of interest, monocytes have to receive stimulatory signals that activate them. The analysis of the changes in monocyte subpopulations reveals a significant difference by arm at 6 months in non-classical monocytes, including intermediate cells that have inflammatory properties.

The balance between monocyte subsets is disrupted after HIV infection, and the number and function of these cells are not completely restored even after long-term ART (13–18). Clearly, monocyte-triggered inflammation plays a main role in fighting a number of infections, and alterations in monocyte functionality that causes changes in their relative proportion influence their capacity to be mobilized, migrate, differentiate into macrophages and trigger either innate or immune responses.

As for T-cell activation, inflammation in response to a phenomenon could be only transient since the immune system tries to reach a new balance, which could explain, at least in part, the changes in monocyte populations that were observed at time 6, but to a less extent at time 12 in the DTG/3TC group. Thus, during HIV infection as well as in its treatment, proportions of monocyte subgroups (classical, intermediate, and non-classical) can provide information regarding the level of residual immune dysfunction and associated risk of serious non-AIDS events (19, 20).

Concerning cardiovascular risk, persistent inflammation and immune activation have been hypothesized to promote atherosclerosis in PLWH (21, 22), as revealed by the measure of carotid intima-media thickness (23). A longitudinal study conducted on 50 PLWH showed that after 24 months of observation, a higher proportion and absolute levels of baseline non-classical monocytes were correlated with an increased carotid artery intima-media thickness at bifurcation, but not at the common carotid artery (24). Another longitudinal study conducted by the same group on 78 PLWH confirmed these initial findings showing that the percentage of non-classical monocytes and plasma MCP-1 levels were independently associated with a coronary artery calcium progression at 24 months, measured by computed tomography examination (25). Of note, none of our patients developed cardiovascular disease events over the year of observation, so we were unable to test this hypothesis.

Concerning IL-6 trajectories, our results are consistent with those of a recent meta-analysis of all published data (including both real-world and randomized studies) showing inconclusive results regarding the level of inflammation after switching to DTG/3TC compared to those seen with 3DRs (12). In particular, the only two randomized clinical trials that included participants who were switched to DTG/3TC (TANGO and SALSA) showed conflicting results. In the SALSA trial, a benefit of DTG/3TC *vs.* 3DRs for soluble CD14 (sCD14) as well as for IL-6 was seen at week 48. In contrast, the TANGO study reported a significant 16% increase in IL-6 and a 3% reduction in sCD14 after 48 weeks of the switch from tenofovir alafenamide-based 3DR to dolutegravir and lamivudine, compared to participants who were kept on the 3DR, which was also appreciated after 144 weeks.

In a real-world study evaluating inflammatory biomarkers, median sCD14 significantly decreased 48 weeks post-switch to DTG/3TC, while other biomarkers remained stable (12). These data are however inconsistent with those of other observational data showing a significant increase in IL-6, high-sensitivity C-reactive protein (hs-CRP), and D-dimer but only starting after 3 years from the date of switching to several 2DRs including DTG/3TC (10). On the one hand, our follow-up was too short to verify this hypothesis; on the other hand, it was possible that changes in plasma levels of this cytokine (which definitely were not of the same magnitude as those observed during COVID-19, for example (26, 27)) were influenced by a number of variables that could be unrelated to the infection.

Our study has several limitations. First of all, as in most small trials, despite randomization (28), we identified imbalances in important predictors of outcome (i.e., AIDS presentation, age, time from HIV diagnosis, and CD4 count nadir), and we tried to minimize possible confounding bias by controlling for these differences by including these factors in our mixed models. Despite this adjustment by regression, we cannot rule out residual or unmeasured confounding (e.g., we did not have data on obesity and smoking, which are known habits related to chronic inflammation) (29, 30). Another key limitation of our analysis is that virological aspects (other than HIV RNA) and in particular the viral reservoir were not investigated. Indeed, viral replication also largely occurs in lymphoid tissues or the central nervous system and other viral reservoirs where low concentrations of antiretroviral drugs are typically seen (31, 32). We believe that our original question is clinically relevant as a decrease in the number of drugs used might have a major impact on viral reservoirs in tissue. Moreover, the follow-up in our trial was only 48 weeks, and over such a short time of observation, it is unlikely to be sufficient to ascertain more complex and durable changes in the immune compartment that could occur in tissue differently from blood. Finally, although our findings are key in light of the fact that dual regimens will be increasingly used in clinical practice, we only have data for DTG/3TC, and we cannot be sure that results are applicable to 2DRs in general. Lastly, although a large number of models have been performed (10 separate regressions), these were conceived *a priori* before seeing the data, and therefore, we did not believe that there was a need for correcting the significance p-value threshold to account for inflation of type I error.

However, this analysis has several strengths. First of all, our analysis confirms that in the rigorous context of a randomized comparison in PLWH with ≤50 copies/mL, after 1 year of therapy, there is little evidence for a difference in immune response when comparing DTG/3TC with B/F/TAF. In addition to randomization, there is the fact that although this study was open-label, all laboratory analyses were conducted without knowing the treatment received by the patients. Moreover, our data could provide a glimpse of what is happening in the lymphoid tissues. Indeed, we observed changes in different blood cell populations that play a key role in triggering or maintaining the immune response, including inflammation. There are two main aspects to consider about this. First, the number of cells in blood represents just a small proportion (approximately 2%) of those belonging to innate or acquired immunity. Thus, even a minimal change in this compartment could be a signal for a much larger change at the level of a number of immune tissues.

In conclusion, our study showed no evidence for a difference in absolute CD4 and CD8 T-cell counts and CD4/CD8 ratio trajectories over 12 months between DTG/3TC and B/F/TAF after the switch. However, the switch to DTG/3TC was associated after 6 months with statistically higher levels both in CD4+ and CD8+ T lymphocytes with markers related to terminal differentiation and exhaustion and in non-classical monocytes, a population of cells that has been recently associated with endothelial dysfunction. Further studies investigating a wider range of dual antiretroviral combinations, longer follow-up, and the power to detect differences in harder clinical outcomes are needed to confirm the clinical impact of our findings.

## Author’s note

Preliminary results were presented at the 30th HIV Glasgow, October 23–26, 2022. Abstract n. P099 published in *Journal of the International AIDS Society* 2022,25(S6):e26009.

## Data availability statement

The raw data supporting the conclusions of this article will be made available by the authors, without undue reservation.

## Ethics statement

The studies involving humans were approved by the local Ethical Committee with Authorization Number AOU 0010923/19 on 12/04/2019 and AIFA Authorization Number AIFA/SC/P/33830 on 25/03/2019. EudraCT Number 2018-003458-26. The studies were conducted in accordance with the local legislation and institutional requirements. The participants provided their written informed consent to participate in this study.

## Author contributions

AC: Conceptualization, Data curation, Formal Analysis, Funding acquisition, Investigation, Methodology, Project administration, Resources, Software, Supervision, Validation, Visualization, Writing – original draft, Writing – review & editing. AC-L: Conceptualization, Data curation, Formal Analysis, Investigation, Methodology, Software, Supervision, Validation, Visualization, Writing – original draft, Writing – review & editing. MMa: Data curation, Investigation, Methodology, Software, Writing – original draft. AP: Data curation, Investigation, Methodology, Writing – original draft. AN: Data curation, Investigation, Methodology, Writing – original draft. SB: Data curation, Investigation, Methodology, Writing – original draft. DL: Data curation, Investigation, Methodology, Writing – original draft. RB: Data curation, Investigation, Methodology, Writing – original draft. LF: Data curation, Investigation, Methodology, Writing – original draft. LG: Data curation, Investigation, Methodology, Supervision, Writing – review & editing. BB: Methodology, Project administration, Resources, Writing – original draft. ER: Methodology, Project administration, Resources, Writing – original draft. GN: Methodology, Project administration, Resources, Writing – original draft. JM: Writing – review & editing, Writing – original draft. MMe: Data curation, Investigation, Writing – original draft. GC: Data curation, Investigation, Writing – original draft. MG: Data curation, Investigation, Writing – original draft. GO: Data curation, Investigation, Writing – original draft. VB: Data curation, Investigation, Writing – original draft. GG: Conceptualization, Investigation, Methodology, Supervision, Writing – review & editing. CM: Conceptualization, Data curation, Formal Analysis, Funding acquisition, Investigation, Methodology, Project administration, Resources, Supervision, Validation, Writing – original draft, Writing – review & editing.

## Supplementary material

Supplementary Figure 1 Box-plots of primary endpoint CD4, CD8 count and CD4/CD8 ratio and adjusted prediction with 95% CI from fitting the mixed linear model.

Supplementary Figure 2 Box-plots of monocytes and IL-6 and adjusted prediction with 95% CI from fitting the mixed linear model.
