# Supplementary material for: Evaluating immunological and inflammatory changes of treatment-experienced people living with HIV switching from first-line triple cART regimens to DTG/3TC vs. B/F/TAF: the DEBATE trial
Source: Front Immunol. 2023 Oct 16;14:1279390. doi: 10.3389/fimmu.2023.1279390 (PMC10613634; doi:10.3389/fimmu.2023.1279390)
Supplement: Supplementary file 1 [file DataSheet_1.docx]

**Supplementary Annex 1. Exclusion criteria**

Participants who met ***any*** of the following exclusion criteria were not eligible for the trial: those with i) chronic hepatitis B; ii) those judged by the study investigators to currently use alcohol or other substances which could potentially interfere with the participant’s study compliance; iii) those either with known hypersensitivity to B/F/TAF FDC tablets, DTG and 3TC, their metabolites, or formulation excipients; iv) those with an active, serious infection (other than HIV-1 infection) requiring parenteral antibiotic or antifungal therapy within 30 days prior to Day 1; or v) those with any other clinical condition or prior therapy that, in the opinion of the investigators, would make the participant unsuitable for the study or unable to comply with protocol requirements.

### **Supplementary Annex 2. Biomarker Testing /Biomarker Samples**

Blood collection and isolation of mononuclear cells

All laboratory analyses were conducted in a blind manner, without knowing the name and the arm of the patients, but only a code. For each participant and at all time points, enrolment (T0) week 24 (T6) and week 48 (T12), we collected peripheral blood in order to analyse in detail: i) the phenotype of T and B lymphocytes, NK cells, and monocytes by polychromatic flow cytometry; ii) to analyse the capability of peripheral blood cells to simultaneously produce different cytokines upon *in vitro* stimulation; iii) to measure plasma levels of 11 pro- and anti-inflammatory molecules.

For these studies, up to 30 mL of blood were collected in vacuettes containing ethylenediamine-tetraacetic acid (EDTA), and blood was immediately processed. Isolation of peripheral blood mononuclear cells (PBMC) was performed using ficoll-hypaque according to standard procedures. PBMC were then vitally stored in different aliquots at the concentration of 5-10 million cells/mL in liquid nitrogen, in foetal bovine serum (FBS) supplemented with 10% dimethyl sulfoxide. Plasma was collected, centrifuged twice, and stored at −80°C until use. Measurements were taken from individual patients; in the case of plasma, each measurement was performed in duplicate and only the mean was considered.

Immunophenotype of peripheral blood mononuclear cells

Thawed PBMC were washed twice with RPMI 1640 supplemented with 10% FBS and 1% each of L-glutamine, sodium pyruvate, nonessential amino acids, antibiotics, 0.1 M HEPES, 55 μM β-mercaptoethanol and 0.02 mg/ml DNAse. For the detailed analysis of T, B, NK cells and monocyte phenotype, PBMC were counted and up to 0.5 million PBMC were stained with different mAbs, incubated, lysed and analysed by flow cytometry, according to the state of the art methodology (11) by using different combinations of directly conjugated mAbs recognizing: CD3, CD4, CD8, CD14, CD16, CD19, CD38, CD45RA, CD197, PD-1 (CD279), CD95, CD57, CD27, CD28, CD80, CD86, HLA-DR, IgM, IgD, HLA-DR. All mAbs were from Becton Dickinson (San José, CA), Beckman Coulter (Brea, CA) or Biolegend (San Diego, CA) and were used after adequate titration. The panel of mAbs was designed to identify different subsets of CD4+ and CD8+ T cells, such as: naïve (N, that are CD45RA+CCR7+), central memory (CM, CD45RA-CCR7+), effector memory (EM, CD45RA-CCR7-,) transitional memory (TM, CD45RA-CCR7-CD27+CD28+), and terminally differentiated (EMRA, CD45RA+CCR7-), exhausted or senescent (CD57+PD1+) and T stem cell memory (CD45RA+CCR7+CD27+CD28+CD95+) lymphocytes. We measured also the main populations of B cells, such as naïve (IgD+IgM+CD27-CD24+), memory switched (IgD-IgM-CD27+CD24-CD38-), memory unswitched (IgD+IgM+CD27+CD24-), transitional (IgD-IgM-CD27-CD24+CD38+), plasmablasts (IgD-IgM-CD27+CD38++); then, natural killer (NK) cells (CD16+CD56+) and monocytes (classical: CD14+CD16- or nonclassical: CD14+CD16+) were measured (12). A minimum of 100,000 cells per sample were acquired on a Cytoflex LX cytometer (Beckman Coulter) and analyzed as described (13).

In vitro stimulation and intracellular cytokine staining

For functional assays on cytokine production by T cells, thawed isolated PBMCs were stimulated for 16 hours at 37°C in a 5% CO_2_ atmosphere with anti-CD3/CD28 (1 μg/mL) in complete culture medium (RPMI 1640 supplemented with 10% FBS and 1% each of L-glutamine, sodium pyruvate, nonessential amino acids, antibiotics, 0.1 M HEPES, 55 μM β-mercaptoethanol) For each sample, at least 2 million cells were left unstimulated as negative control, and 2 million cells were stimulated. All samples were incubated with a protein transport inhibitor containing brefeldin A (GolgiPlug, Becton Dickinson) and previously titrated concentration of CD107a-PE. After stimulation, cells were stained with LIVE-DEAD Aqua (ThermoFisher Scientific, Eugene, OR) and surface mAbs recognizing CD3, CD4 or CD8 molecules (from Biolegend) (14). Cells were washed with staining buffer, fixed and permeabilized with the cytofix/cytoperm buffer set (Becton Dickinson). Cells were next stained with previously titrated mAbs recognizing intracellular IL-17 (conjugated with Brillant Violet 421), TNF (BV 605), IFN-γ (FITC), IL-2 (APC), or granzyme-B (BV 421; all these mAbs were from Biolegend). Then, a minimum of 100,000 cells per sample were acquired on an Attune NxT acoustic cytometer (ThermoFisher), as described (15).

Quantification of cytokines and other soluble molecules

We used the Luminex system for the quantification of 11 pro- and anti-inflammatory molecules in the same sample and kits from R&D System (Minneapolis, MN), as previously reported (16). All these biomarker tests were performed at the Laboratory of Immunology (Department of Medical and Surgical Sciences for Children & Adults, University of Modena and Reggio Emilia) and, like for all the other assays described above, laboratory personnel were blind to participants arm allocation and outcomes at the time of performing the tests.

**Supplementary Annex 3**. **The fit of the regression model and checking of assumptions**

Log-transformation was used to achieve symmetry in the distributions of outcome variables and we also checked the assumptions of homogeneity of variance of residuals. To keep the model simple we fitted a linear predictors of the covariates. Of note, chance imbalance was unexpected and therefore the analysis plan in the protocol did not mention multivariate analyses (i.e. the mixed linear model).
