# Supplementary figures and images for: Evaluating immunological and inflammatory changes of treatment-experienced people living with HIV switching from first-line triple cART regimens to DTG/3TC vs. B/F/TAF: the DEBATE trial
Source: Front Immunol. 2023 Oct 16;14:1279390. doi: 10.3389/fimmu.2023.1279390 (PMC10613634; doi:10.3389/fimmu.2023.1279390)

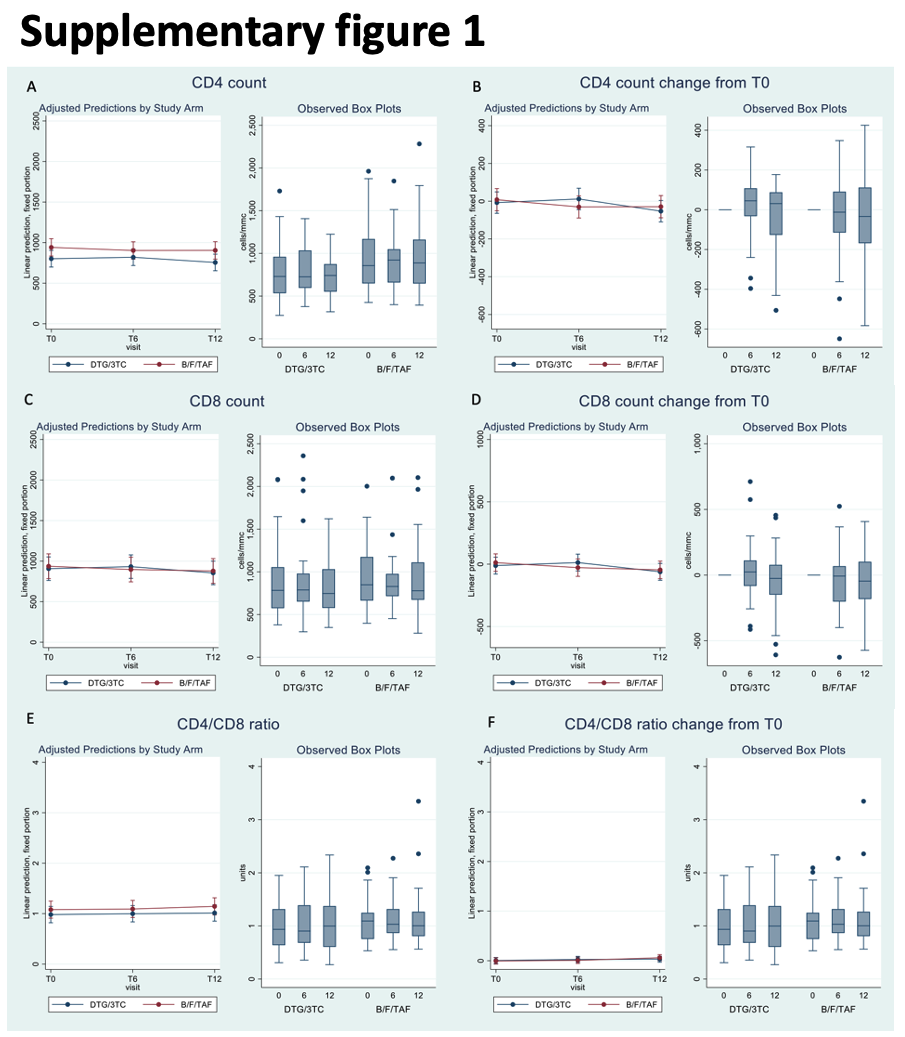

Supplement: Supplementary Figure 1 — Box-plots of primary endpoint CD4, CD8 count and CD4/CD8 ratio and adjusted prediction with 95% CI from fitting the mixed linear model. [file Image_1.png]

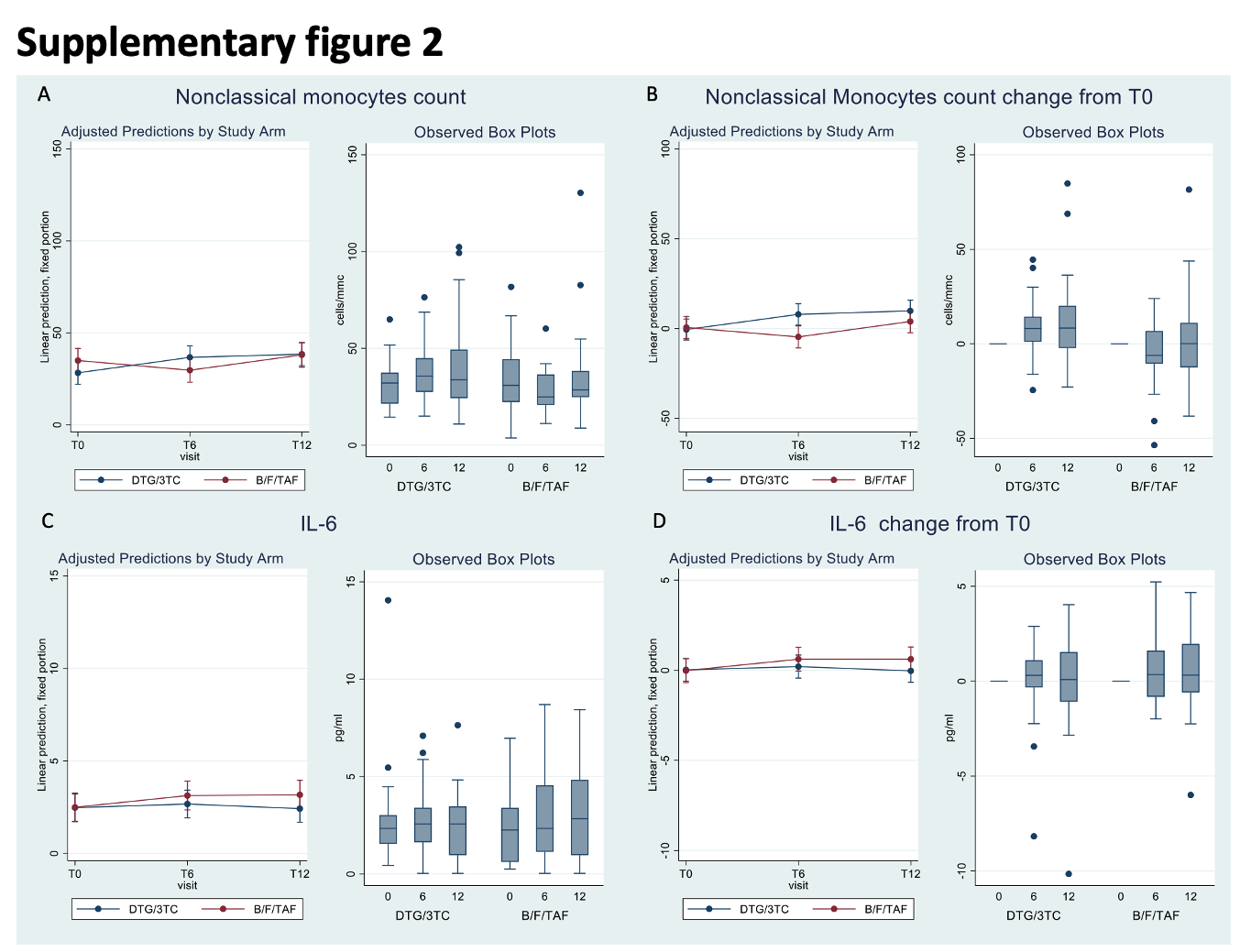

Supplement: Supplementary Figure 2 — Box-plots of monocytes and IL-6 and adjusted prediction with 95% CI from fitting the mixed linear model. [file Image_2.png]
